# Airborne particulate matter

**DOI:** 10.1098/rsta.2019.0319

**Published:** 2020-09-28

**Authors:** Roy M. Harrison

**Affiliations:** Division of Environmental Health and Risk Management, School of Geography, Earth and Environmental Sciences, University of Birmingham, Birmingham B15 2TT, UK

**Keywords:** airborne particulate matter, PM_2.5_, PM_10_, particle number, ultrafine particles, source apportionment

## Abstract

Airborne particulate matter (PM) is a pollutant of concern not only because of its adverse effects on human health but because of its ability to reduce visibility and soil buildings and materials. It can be regarded as a suite of pollutants since PM covers a very wide range of particle sizes and also has a diverse chemical composition. Historically, much of the PM arose from coal burning and was measured as black smoke. However, in the second half of the twentieth century in developed countries, there was a reduction in black smoke emissions from coal burning and PM steadily became dominated by carbonaceous particles from road traffic exhaust and the secondary pollutants, ammonium salts and secondary organic carbon. This is exemplified by the composition of fine particles (referred to as PM_2.5_) as measured in London, Delhi and Beijing. Steadily, as control strategies have addressed the more tractable sources of emissions, so sources previously regarded as unconventional have emerged and have been seen to make a significant contribution to airborne PM concentrations. Among these are non-exhaust particles from road traffic, cooking aerosol and wood smoke. The particle size distribution of airborne PM is hugely diverse, ranging from newly formed particles of a few nanometres in diameter through to particles of tens of micrometres in diameter. There has been a great deal of interest in ultrafine (nano) particles because of suspicions of enhanced toxicity, and as traffic emissions decrease as a source, so regional nucleation processes have become much bigger relative contributors to particle number, but not mass.

This article is part of a discussion meeting issue ‘Air quality, past present and future’.

## 1. Introduction

Airborne particulate matter (PM) is a pollutant of great importance which presents many challenges. Its significance lies particularly within two areas. Firstly, it is the pollutant having by far the largest impact upon public health. This is clearly elaborated by other contributions to this volume, and according to the Global Burden of Disease study [1], it ranks very highly among the avoidable causes of non-communicable diseases. PM is also important because it both absorbs and reflects solar radiation and therefore affects climate [2]. Absorption of incoming solar radiation by components such as black carbon, which are strongly absorptive, causes local heating of the atmosphere while more reflective particles such as ammonium sulfate reflect sunlight back to space and have a net cooling effect at the surface. However, there are also secondary effects concerned with cloud formation, and the number density of particles referred to as cloud condensation nuclei has a profound influence upon the albedo of clouds and hence upon surface air temperatures [3]. Such effects are not the focus of this paper which will address primarily issues concerned with the size distribution, chemical composition and sources of airborne PM. It will consider emerging contributors to PM concentrations before focussing on airborne nanoparticles and considering possible impacts upon future concentrations.

Airborne particles present great complexity because unlike atmospheric trace gases which have the same chemical and physical properties wherever they occur, airborne particles are in reality a suite of pollutants varying in particle size and chemical composition on a range of temporal and spatial scales. Airborne particles can be both directly emitted, referred to as primary, or formed within the atmosphere from the condensation of trace gases, referred to as secondary particles. The smallest such particles arising from gas-to-particle conversion processes are 1–2 nm in diameter. The largest airborne particles are in excess of 100 µm in diameter but have a rather short atmospheric lifetime due to high gravitational settling speeds. The air quality guidelines and standards applicable to PM are framed in terms of two health-relevant fractions. The first is referred to as PM_2.5_, which describes particles measured by mass which pass a sampling inlet with a 50% cut-off efficiency at 2.5 µm. They are, therefore, in effect all particles smaller than 2.5 µm. The other metric, PM_10_, describes particles measured by mass passing a sampler inlet with a 50% efficiency cut-off at 10 µm. It therefore includes the mass of all particles smaller than 10 µm and consequently includes PM_2.5_. The other relevant definition is that of ultrafine particles, generally defined as particles with one dimension smaller than 100 nm (0.1 µm). These are often also referred to as nanoparticles because of their nanometre dimensions.

**Figure 1.**
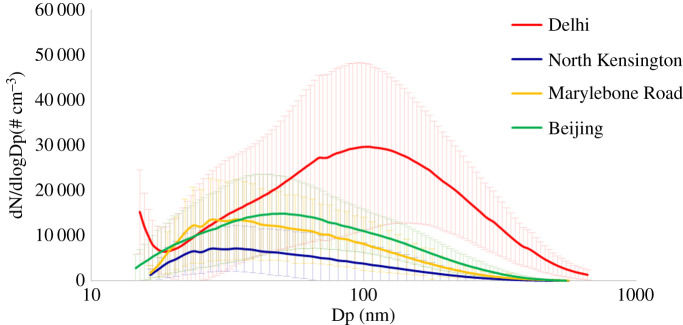
Average particle number size distributions measured in London (North Kensington and Marylebone Road), Beijing and Delhi. The shading represents one standard deviation. (Online version in colour.)

**Figure 2.**
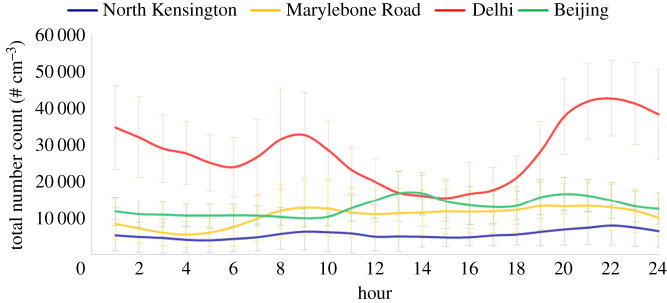
Average diurnal variation of particle number counts at the sites in London (North Kensington and Marylebone Road), Beijing and Delhi. The error bars represent one standard deviation. (Online version in colour.)

Harrison *et al*. [4] have described the size distribution of particles and how the characteristics of the size distribution can look very different according to whether it is expressed in terms of particle number, surface area or volume/mass. Most airborne particles by number are typically smaller than 100 nm diameter and hence the peak abundance of particles in the urban atmosphere is often at a size of around 20–30 nm, and in European and North American cities, typically 80–90% of particles by number are smaller than 100 nm. However, when the size distribution is transformed into a surface area distribution, the majority of the surface area is associated with particles in the 0.1–1 µm size range referred to as the accumulation mode. Nanoparticles contribute only a relatively small amount of the total surface area. When the distribution is converted to a volume distribution or a mass distribution (if the density is known), two modes typically appear; one in the 0.1–1 µm range referred to as the fine mode and one in the 1–10 µm range referred to as the coarse mode. The minimum between the two most typically lies at around 1 µm, but the sub-division at 2.5 µm used by regulatory agencies to define the PM_2.5_ metric provides an approximate sub-division of the fine fraction. As a general observation, fine fraction particles arise predominantly from gas-to-particle conversion processes within the atmosphere or from emissions from high-temperature processes such as vehicle exhaust or industrial combustion. On the other hand, coarse fraction particles are more typically associated with mechanical break-up through abrasion or wind-driven processes such as soil resuspension or the creation of sea spray by breaking waves. Figure 1 shows average particle number size distributions measured by a Scanning Mobility Particle Sizer, which separates particles on the basis of giving them charge and then measuring their mobility in an electric field, from air sampling campaigns in London, Beijing and Delhi. These are winter campaign data which do not reflect the major seasonal changes in particle concentrations which are seen in both Delhi and Beijing, and rather less in London. The lowest concentrations are seen at the London North Kensington site which is an urban background location in central London. The Marylebone Road site is at roadside and shows a broadly similar size distribution but an appreciably higher average concentration. The particles from Beijing are greater in both number and size while those in Delhi are far greater in number with a much larger modal diameter close to 100 nm. It, therefore, appears that the number concentration and modal diameter tend to scale with the degree of pollution of the city, with Delhi showing by far the highest pollution levels and London the lowest. The trend in diameter may reflect different predominant emission sources or may be the result of particle growth in the atmosphere. In more polluted atmospheres, particles grow more rapidly by coagulation which depends upon particle number concentrations and by condensational growth largely due to atmospheric oxidation processes creating species of low volatility which condense onto existing particles causing them to grow [5]. Figure 2 shows typical diurnal variations of particle number counts from London, Beijing and Delhi. Just discernable in the Marylebone Road and North Kensington data are the influences of road traffic emissions, and most notably the morning rush hour, on number concentrations, while in Beijing, the highest concentrations are seen in the early afternoon. This is due to two major differences from London. Firstly, the light-duty vehicle fleet in Beijing is wholly gasoline fuelled which leads to much lower emissions of particles than the diesels which contribute a large part of the light-duty fleet in London. In London, heavy-duty vehicles can move within the city at all times of day and night, whereas in Beijing the heavy-duty vehicles are restricted to the nighttime hours. The early afternoon peak in Beijing is almost certainly the result of new particle formation through regional nucleation processes referred to later in this article [6,7]. The diurnal variation in Delhi is suggestive of a major influence of road traffic especially at nighttime when the heavy-duty vehicles enter the city. There is, however, also a strong diurnal variation of atmospheric boundary layer mixing depths in Delhi with a much shallower mixed layer at nighttime which is no doubt a contributor to the very high concentrations seen at this time of the day.

## 2. Major sources of airborne particulate matter

Particles sampled over open ocean areas show a very major contribution from sea salt as well as oxidation products of trace gases such as dimethylsulfide which are released from the oceans. However, over land there tends to be a rather different composition dominated by primary and secondary components deriving from anthropogenic emissions [8,9]. Table 1 indicates some of the major categories of particles together with their main chemical components and predominant sources. Airborne PM is hugely diverse so this list is by no means exhaustive and there are many trace element components which are not listed in the table. Road traffic exhaust has for many years been a major contributor, although this is now declining in most of the developed world [10]. Road traffic exhaust often dominates the particle number concentrations and figure 1 shows a clear difference between the roadside location at Marylebone Road and the background North Kensington which is attributable almost wholly to emissions from traffic on Marylebone Road. The particles typically arise predominantly from older diesel vehicles, although with advances in technology, the contributions from gasoline are becoming more notable in cities like London, and in North America gasoline is dominant [11]. The vehicle exhaust particles are comprised very largely of elemental carbon, referred to as black carbon and organic compounds, which derive both from unburnt fuel and from lubricating oil vapourized within the engine [12]. In London, diesel exhaust is the main source of elemental carbon, while in Beijing coal burning is a major source of particles comprised of elemental carbon and organic compounds [13]. Sea salt, with major components sodium, magnesium and chloride, makes major contributions at coastal locations but can still be seen hundreds of kilometres inland. Chloride ion also arises from neutralization of HCl vapour generated in the combustion of coal and some plastics, and makes a notable contribution in Delhi [14]. The ammonium ion derives from ammonia gas which comes largely from agriculture and is the main neutralizing species for HCl, and for nitric and sulfuric acids which arise, respectively, from the oxidation of nitrogen dioxide and sulfur dioxide, with the production of ammonium nitrate and ammonium sulfate which are major constituents of airborne particles. There are numerous sources which emit organic matter, most notable among them are biomass burning, both of wood and crop residues, and cooking [15]. Oxidation of organic vapours in the atmosphere leads to the formation of secondary organic matter which can be difficult to differentiate from primary emissions but appears to contribute a large proportion of atmospheric organic matter, amounting to 19% in a London winter campaign [16], and 16–65% in datasets from China [15]. Within this, there is a large secondary contribution from the oxidation of biogenic volatile organic compounds emitted by terrestrial vegetation, especially in summer when emissions of BVOC are highest. Differentiation of the contributions of anthropogenic and biogenic precursor VOC is challenging, but estimates of the annual average contribution of biogenic sources to SOA in China are 35%, and for southern China, 65–85% [15]. Changes in NO*_x_* emissions will impact upon SOA formation, as SOA yields are higher as NO*_x_* concentrations decline [17] which needs to be accounted for in developing policy.

**Table 1.** Major sources of particulate matter.

category	main chemical components/source
road traffic exhaust	elemental (black) carbon, organic compounds
sea salt	sodium, magnesium, chloride
ammonium	ammonia (largely from agriculture)
nitrate	oxidation of nitrogen dioxide
sulfate	oxidation of sulfur dioxide
primary organic matter	wood smoke, coal smoke, cooking, etc.
secondary organic matter	oxidation of organic vapours
dust and soil	silicon, aluminium, calcium

**Figure 3.**
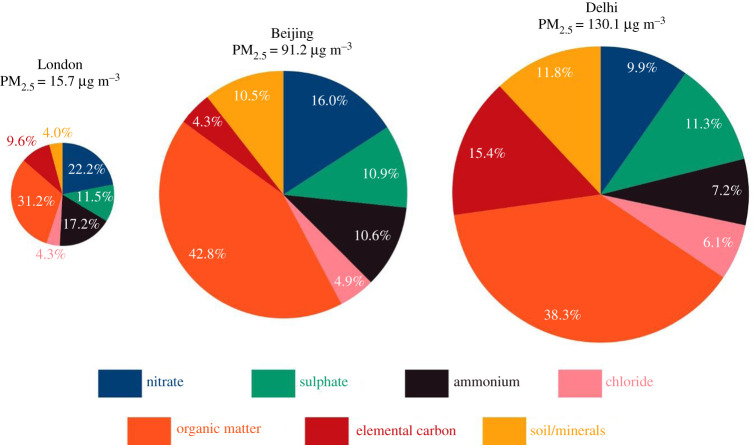
Major chemical component composition of PM_2.5_ collected during winter campaigns in London (North Kensington), Beijing and Delhi. (Online version in colour.)

The atmosphere also contains wind-blown dusts and soils which tend to reflect local geological conditions, with major components of silicon, aluminium and calcium typically, although these dusts can become contaminated with trace metals, for example as in road dust. As can be seen from figure 3, the major component composition of PM_2.5_ particles in London, Beijing and Delhi is not greatly different. There is far greater difference in the average mass concentrations, all of which were measured in winter sampling campaigns in these cities and do not represent the annual means which are somewhat lower. These pie charts clearly illustrate the huge importance of the organic matter and ammonium nitrate and sulfate as typically dominant constituents.

Chemical composition in the form of that in figure 3 does not reveal very full information on the sources of airborne particles. However, if a larger number of chemical constituents are measured on a large number of individual air samples, receptor modelling methods can be used to infer the sources of particles [18]. The term receptor modelling refers to the use of air quality data to infer the sources responsible for measured pollution levels and is the complement of dispersion modelling and chemistry-transport modelling which take the known emissions and disperse and chemically react them in the atmosphere to predict airborne concentrations. Receptor modelling of airborne particles depends upon an assumption of mass conservation as in the equation below:

$$C_{i}=\overset{i}{\underset{\;}{\sum }}f_{i,j}g_{j},$$

where *C_i_*, airborne concentration of a component, *i*; *f_i,j_*, mass fraction of component *i* in particles from source, *j*; *g_j_*, mass of particles from source *j* in an air sample.

**Figure 4.**
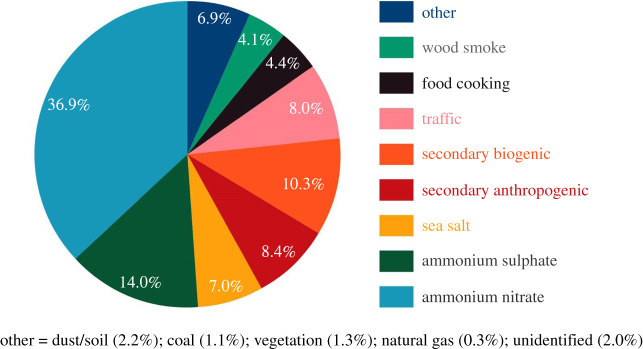
Source contributions to PM_2.5_ at North Kensington (%) derived from the application of a Chemical Mass Balance model (data from [16]). (Online version in colour.)

There are two main approaches used for receptor modelling. Multivariate statistical methods such as Positive Matrix Factorization make no *a priori* assumptions about sources and give a quantitative identification of those constituents which covary in time generating the chemical profiles of source-related factors, which with suitable intuition and knowledge, can be used to infer the sources. The other approach of Chemical Mass Balance modelling approaches the problem from the other end. It uses chemical profiles of known sources as an input and fits the measured chemical data with the best linear combination of source profiles so as to explain the measured concentration of each chemical constituent. This can in theory be carried out on a single air sample, but in practice better results are obtained by the inclusion of multiple samples. An example of the application of the Chemical Mass Balance model to PM_2.5_ from the London North Kensington site [16] is shown in figure 4. A notable weakness of all receptor modelling methods is the dependence upon the assumption that the chemical profiles of sources are conserved between source and receptor points. In practice, such profiles are subject to chemical change, although this is less of an issue for urban samples collected close to sources than for more remote sites.

## 3. Emerging sources of airborne particulate matter

In less developed countries, airborne particles tend to be dominated by very familiar sources. Older and poorly maintained road vehicles are a major source of exhaust emissions and uncontrolled or poorly controlled combustion of fossil and biomass fuels as well as open burning of refuse are typically major contributors, both of primary particles and precursor gases such as HCl and VOC. Many less developed countries are also in drier parts of the world and consequently wind-blown dusts often of largely natural origin can make major contributions to PM concentrations [19]. However, in the more developed world, such sources are generally under far better control, and as they have become more controlled, so other less familiar sources are beginning to emerge as important. Four such sources will now be considered.

### (a) Non-exhaust emissions from road traffic

Road vehicles marketed in Europe and many other parts of the world have to meet emissions standards referred to as the Euro standards, or national equivalents of the same. Since the introduction of the Euro 5 standard for light-duty vehicles and Euro 6 for heavy-duty vehicles, the requirements for very low particle number emissions can only be met by the fitting of diesel particle filters. More recent regulations are also requiring the use of gasoline particle filters on gasoline direct injection engines. As a consequence, there has been a marked decline in vehicle-emitted particles from road traffic both in terms of number and mass [10]. In such a situation, non-exhaust emissions become a bigger proportion of the total emissions from road traffic, and according to estimates from the UK National Atmospheric Emissions Inventory, the non-exhaust emissions now well exceed the exhaust emissions both in the case of PM_10_ and of PM_2.5_ [20]. Non-exhaust emissions are made up predominantly from abrasion particles of brake dust deriving both from the attrition of the disc and the pad, tyre dust, road surface abrasion particles, and also resuspended road surface dusts which are not currently included in the UK inventory [21,22]. The latter arise predominantly as a result of shear forces at the road surface created by wheels passing over the road and also resuspension due to turbulence occurring in the wake of the passing vehicle. A recent study from Delhi attributes a very large proportion (70%) of road traffic emissions of PM_10_ to particle resuspension [23] although the algorithm used to estimate resuspension is controversial [24], and the contribution to PM_2.5_ mass is probably very much smaller. Currently, none of these particle sources is subject to legislative control, but there is a good deal of research on the control of brake wear particles which is probably the most tractable of the emission problems. The regular cleaning of road surfaces can reduce the particle resuspension problem but has a very limited time duration of effect, and there are also dust suppressant materials which can be sprayed onto the road surface to limit the ability of particles to enter the atmosphere through resuspension [25].

### (b) Cooking aerosol

Major advances in understanding the sources of airborne particles have arisen as a result of the high time resolution data generated by aerosol mass spectrometers (AMS) [26]. The AMS measures the mass spectral properties of non-refractory sub-micrometre airborne particles sampled directly from the atmosphere. Application of Positive Matrix Factorization to the mass spectral data allows the identification of source-related components of the particles and has revealed a component with a chemical signature quite close to that of cooking oils and a diurnal variation with a small peak around midday and a large evening peak [27]. This is attributed to cooking organic aerosol which has also been quantified through chemical mass balance modelling using chemical tracers for cooking [16]. There is evidence that many past studies using the AMS method may have over-estimated airborne concentrations of cooking organic aerosol [28], but even allowing for this over-estimation, concentrations in the atmosphere are appreciable, and work in the USA has shown highly elevated concentrations within the vicinity of major commercial restaurants [29].

### (c) Domestic wood combustion

Some western countries saw an increase in the burning of solid fuels and especially biomass as a result of fuel poverty caused by the economic recession. However, in the UK, an increased use of biomass fuels, and especially wood, has resulted from a fashion for installing wood-burning stoves or using open fireplaces within homes. This appears to be associated more with the aesthetic pleasures of a fire rather than as a primary means of heating. According to the UK National Atmospheric Emissions Inventory, this has caused a marked upward trend in emissions from domestic wood combustion especially when expressed as a percentage of the total primary emissions of PM_2.5_. The estimate for 2012 is that biomass sources, of which domestic wood burning is the largest, contributed 25% of total primary PM_2.5_ emissions [30]. The UK Air Quality Expert Group, a government advisory committee, compared emissions of PM_2.5_ from woodstoves operating at the limits set by the Clean Air Act and the EU Eco-Design Directive with emissions from diesel vehicles running at their upper limit, and the emissions from a single woodstove far exceed those from a modern diesel passenger car or heavy goods vehicle [30]. This appears to be a widespread problem affecting all areas, even including cities where clean air legislation attempts to limit the use of fuels such as wood. In the UK, the perception that biomass fuels are renewable has led to their incentivisation through the Renewable Heat Incentive, which has thus far influenced mainly the installation of larger combustion plant rather than domestic stoves, but seems likely to impact adversely upon local air quality. In China and India, the burning of crop residues can be a very major source of PM [31] which is subject to regional transport, affecting cities at some distance from the location of the combustion. This source has been very much reduced in the Beijing area as part of the 5 year Clean Air Action Plan which has led to significant improvements in urban air quality [32]. A further point noted by AQEG [30] is the large semi-volatile organic content of particles from sources such as wood burning, which are often not adequately accounted for in emissions inventories due to poorly designed sampling protocols. These contribute both to the mass of primary particles and to subsequent secondary particle formation [33].

## 4. Secondary particles

Emissions of sulfur dioxide have decreased hugely in western countries over the past decades and airborne concentrations of sulfur dioxide have reduced in a proportionate manner. On the contrary, concentrations of sulfate appear to be nonlinearly related to SO_2_ emissions and have not fallen proportionately in Europe [34], or North America [35]. Oxides of nitrogen emissions have also reduced but far less than those of sulfur dioxide largely because of poor controls applied to the road vehicle fleet which have only recently started to impact on NO*_x_* emissions. Nitrate has not shown a commensurate reduction [35]. Consequently, secondary nitrates typically represent the largest single component of PM_2.5_ in countries such as the UK [36]. A factor in the resistance of ammonium nitrate concentrations to respond to mitigation measures is the fact that emissions of ammonia, largely from agriculture, have been reduced little if at all in recent decades and high ammonia concentrations favour the formation of ammonium nitrate particles which otherwise would be liable to dissociate into ammonia and nitric acid gases which would be subject to more efficient dry deposition processes [37].

While pollution by nitrates and sulfates has been well understood for many years, it still presents significant difficulties in chemistry-transport models largely because of a proliferation of mechanisms which are quite hard to differentiate using atmospheric measurements. However, among emerging pollutants, secondary organic aerosol (SOA) is now receiving significant attention. There are no wholly reliable methods of differentiating between primary and secondary organic aerosol, but current estimates suggest that secondary aerosol comprises a major proportion of organic particles. There has been even more difficulty in linking back secondary organic aerosol to specific chemical precursors, although there is fairly good knowledge of the contribution of biogenic precursors such as isoprene and α-pinene as well as anthropogenic emissions of compounds such as toluene to the production of secondary organic particles [16,38]. There have been major reductions in VOC emissions in the UK over past decades but it is unclear whether these have been reflected in a reduction of anthropogenic secondary aerosol. The biogenic precursors are known to make a significant contribution and there seems little prospect of these reducing in the near future. It appears that non-traffic related VOC arising from domestic emissions of solvents and personal care products now contribute substantially to secondary organic aerosol [39].

## 5. Ultrafine (nano) particles

As mentioned earlier, ultrafine are particles usually defined as those with one dimension less than 100 nm. They can be measured by mass and are referred to as PM_0.1_, but this is technically quite difficult. They are far more often measured by number, and since ultrafine particles dominate the number count in most atmospheres (figure 1), the total particle number count is typically used as a surrogate for the ultrafine particle concentration. Emissions inventories have been constructed both in terms of mass and of number. The UK atmospheric emissions inventory uses a simple method to calculate a mass-based inventory by taking an inventory of particles in a larger size range such as PM_2.5_ and using an estimated percentage from each source sector to estimate the PM_0.1_ emissions. On the other hand, TNO in the Netherlands has generated an inventory of ultrafine particle emissions within Europe based upon particle number [40]. Within this inventory, the transport sector contributions about 75% of total particle number emissions with international shipping and diesel road traffic making by far the major contributions. As a result, a map of emissions serves to highlight the major shipping and road traffic routes within Europe. Projections for future years from a 2005 baseline show major reductions for 2020, mainly delivered by reductions in emissions from the transport sector [40]. This is due to the fact that particle number emissions from combustion sources are highly sensitive to the sulfur content of the fuel, and motor fuels have steadily reduced their sulfur content, now less than 10 parts per million, and shipping fuels are progressively reducing sulfur but not yet to the same degree. A notable exception is emissions from jet aircraft for which fuels still contain several hundred parts per million of sulfur, and emissions from major airports are detectable in the atmosphere in a number of major European cities at a considerable distance from the main point of emission [41,42]. Ultrafine particles from road traffic comprise both a soot mode of primarily graphitic carbon with a lesser amount of associated organic matter, and a nucleation mode which is primarily semi-volatile organic compounds condensed on the surface of a very small nucleus of sulfuric acid or inorganic ash derived from engine emissions [5]. The nucleated component has tended in the past to dominate particle number emissions, but the reduction in the sulfur content of road vehicle fuels, which took place in late 2007 in the UK and at similar times elsewhere in Europe, led to a very major reduction in particle number concentrations at roadside locations such as Marylebone Road [43].

In addition to those particles arising from combustion processes, ultrafine particles also arise in the atmosphere from homogeneous nucleation processes. These generally involve the initial formation of sulfuric acid vapour which condenses along with ammonia, amines and water to form new particles which subsequently grow by condensation of organic matter [7]. Mechanisms have also been demonstrated in which both biogenic [44] and anthropogenic hydrocarbons [45] are oxidized to form highly oxygenated molecules (HOMs) which can condense either alone, or in combination with sulfuric acid to form new particles. Such particles can appear over quite large geographical regions simultaneously and are hence often referred to as arising from regional nucleation. The initial formation of sulfuric acid vapour or HOMs depends upon photochemistry, and an analysis of average diurnal profiles of particle number count and of black carbon used as a sensitive tracer for diesel emissions shows marked differences between northern and southern Europe [46]. At sites in northern Europe, there is generally a strong correlation between the diurnal variation of particle number and of black carbon suggesting diesel emissions as being the major source of both constituents. In southern Europe, however, there is typically a further large peak in particle number count in the middle of the day which is not reflected in the black carbon data. This is the result of regional photochemical nucleation and can make a major contribution to airborne concentrations of ultrafine particles [47].

Much of the interest in ultrafine particles arises from the suggestion that they may have enhanced toxicity per unit mass compared to larger particle size fractions [48]. Currently, however, evidence on health impacts is sparse and lacks overall consistency [49], and the relative health impacts of primary nanoparticles from road traffic as compared to secondary particles from regional nucleation is not well understood.

## 6. The future

Road traffic has traditionally been considered as the main culprit for high concentrations of PM_2.5_. However, in cities with modern and well-maintained vehicle fleets, the contribution of vehicle exhaust to PM_2.5_ concentrations is decreasing rapidly due to the use of particle traps on modern vehicles. Although there are minor emissions during trap regeneration, these traps are almost 100% efficient in removing particles from engine exhaust. Consequently, the non-exhaust particles which now dominate the emissions are becoming a much greater concern and centre of attention. Other sources outlined above such as non-exhaust particles from road traffic, domestic wood burning, cooking and secondary organic aerosol are now seen as making important contributions to airborne concentrations of PM_2.5_. Sulfate concentrations have fallen in major western countries and are likely to continue to fall unless there is a reversal of emissions controls on sulfur dioxide, but future declines will be hard won due to the nonlinearity of the relationship between sulfur dioxide and sulfate concentrations. Airborne nitrate is currently the largest single component of PM_2.5_ in many developed countries and work in the UK has shown that its relative contribution increases during air pollution episodes, in this case, represented by 24-h periods where PM_10_ concentration exceeded the EU daily Limit Value of 50 µg m^−3^. Because of the complex formation mechanisms of nitrate, the nonlinearity between NO*_x_* emissions and nitrogen dioxide concentrations and the impacts of ammonia upon ammonium nitrate formation, the reductions in NO*_x_* emissions which are largely due to better controls on road traffic are most unlikely to have a substantial impact on atmospheric nitrate levels which will be far harder to control. The key may well lie in reductions of ammonia emissions, but historically these have been subject to lesser control than the other primary pollutants and there need to be major changes in policy if ammonia concentrations are to reduce to a meaningful degree. Secondary organic aerosol has a large contribution from biogenic precursors which will not change rapidly over time and will only reduce if there is clear attention to possibilities favouring low emitting species of shrubs and trees over the higher emitting species. The links between anthropogenic VOCs and SOA are in general far less clear, and SOA seems to have responded in a relatively minor way to major reductions in VOC emissions which have occurred in the UK. This suggests SOA is unlikely to reduce rapidly in the future. As a consequence of these various changes, there is a huge challenge for countries such as the UK to meet the current WHO air quality guideline for PM_2.5_ of 10 µg m^−3^ as an annual mean.

## Data accessibility

Data supporting this publication are openly available from the UBIRA eData repository at https://doi.org/10.25500/edata.bham.00000435.
